# A Combination of Geniposide and Chlorogenic Acid Combination Ameliorates Nonalcoholic Steatohepatitis in Mice by Inhibiting Kupffer Cell Activation

**DOI:** 10.1155/2021/6615881

**Published:** 2021-05-13

**Authors:** Xin Xin, Yue Jin, Xin Wang, Beiyu Cai, Ziming An, Yi-Yang Hu, Qin Feng

**Affiliations:** ^1^Institute of Liver Diseases, Shuguang Hospital Affiliated to Shanghai University of Traditional Chinese Medicine, Shanghai 201203, China; ^2^Shanghai Key Laboratory of Traditional Chinese Clinical Medicine, Shanghai 201203, China; ^3^Key Laboratory of Liver and Kidney Diseases, Shanghai University of Traditional Chinese Medicine, Ministry of Education, Shanghai 201203, China

## Abstract

The incidence of nonalcoholic steatohepatitis (NASH) is increasing worldwide. Activation of Kupffer cells (KCs) is central to the development of diet-induced NASH. We investigated whether a combination of two active chemical components, geniposide and chlorogenic acid (GC), at a specific ratio (67 : 1), ameliorates diet-induced NASH and the underlying mechanisms involved. C57BL/6J mice exposed to a high-fat and high-cholesterol (HFHC) diet containing cholesterol, choline, and high-sugar drinking water, as well as RAW264.7 cells stimulated with lipopolysaccharide (LPS) were studied. The combination exerted a therapeutic effect on HFHC-induced NASH in mice. Simultaneously, GC was found to reduce the expression of cytokines secreted by hepatic macrophages, including tumor necrosis factor-*α* (TNF-*α*), interleukin-1*α* (IL-1*α*), IL-1*β*, IL-6, monocyte chemotactic protein 1 (MCP-1), and granulocyte-macrophage colony-stimulating factor (GM-CSF). Moreover, GC reduced the number of KCs expressing F4/80. Furthermore, TNF-*α*, inducible nitric oxide synthase (INOS), IL-1*β*, and IL-6 mRNA and TNF-*α* protein expression levels were suppressed upon GC treatment in RAW264.7 cells. Our findings suggest that GC has a strong anti-inflammatory effect in NASH, and this effect can be attributed to the suppression of KC activity in the liver.

## 1. Introduction

With the increasing incidence of obesity, diabetes, and metabolic syndromes, nonalcoholic fatty liver disease (NAFLD) is emerging as one of the most common causes of chronic liver disease. Recently, the acronym metabolic associated fatty liver disease (MAFLD) has replaced the use of NAFLD in literature [1]. NAFLD encompasses a wide spectrum of liver damage, ranging from benign lipid accumulation in the liver (steatosis) to nonalcoholic steatohepatitis (NASH) characterized by fat vacuoles, lobular inflammation, and hepatocyte damage in the form of ballooning and apoptosis [2, 3]. At an advanced stage of NAFLD, patients with NASH are at the risk of developing liver fibrosis, liver cirrhosis, and hepatocellular carcinoma (HCC) [4]. The causes of progression from steatosis to NASH are lipotoxicity, oxidative stress, and inflammation [5]. Although many attempts have been made to find an appropriate molecular target and related chemical drugs to treat NASH, there is currently no effective therapy that has been approved for NASH. Thus, novel and effective therapies to improve the prognosis of NASH are urgently needed. The pathophysiology of NASH is multifactorial and not yet completely understood [6]; however, innate immunity is a major contributing factor in which liver-resident macrophages and recruited macrophages play a central role [7]. Liver-resident Kupffer cells (KCs) initiate inflammation and help recruit blood-derived monocytes; both differentiate into proinflammatory macrophages and further promote NASH progression [8].

The combination of geniposide and chlorogenic acid (GC) has been derived from a traditional Chinese medicine [9] called Qushi Huayu Decoction (QHD) which consists of five herbs, including *Artemisia capillaris* Thunb., *Gardenia jasminoides* Ellis, *Polygonum cuspidatum* Sieb. et Zucc., *Curcuma longa* L., and *Hypericum japonicum* Thunb. It has been used clinically to treat NAFLD for decades in China and has been validated in multiple NAFLD animal models [10, 11]. In our previous study, the principal active component of each Chinese medicinal herb used in QHD was screened at different dosages using uniform design, and hepatic triglyceride (TG) was used as the screening index in NAFLD induced by a high-fat diet (HFD) in mice [12]. The regression equation suggested that the mixture consisting of geniposide from G. jasminoides Ellis (90 mg/kg per day) and chlorogenic acid from A. capillaries Thunb. (1.34 mg/kg per day) exhibited the most powerful inhibition of lipid deposition in NAFLD mice [10, 12]. These previous studies not only proved the efficacy of GC in treating NAFLD but also discussed its pharmacological mechanism. We have also shown that GC ameliorates NAFLD by reducing gut inflammation and improving gut barrier function [9, 11]. The molecular mechanism underlying GC-mediated regulation of hepatic immunity is still poorly understood.

To determine the potential mechanisms of GC action and explore the regulatory effect of GC on KCs in the progression of NASH, we used a high-fat and high-cholesterol (HFHC) (high-trans fat, high-cholesterol, and high-bile-salt feed) diet and high-sugar drinking water to induce NASH in a C57BL/6 mice model and RAW264.7 cells. We focused on changes in the KCs and liver inflammation factors.

## 2. Materials and Method

### 2.1. Drug Preparation and Identification

**Figure 1:**
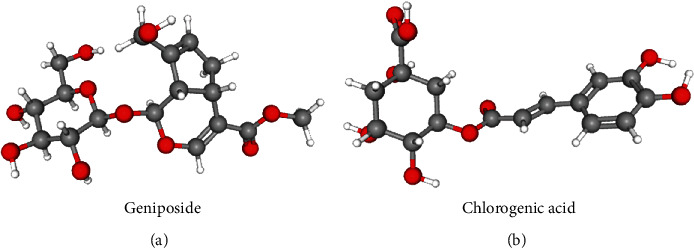
The chemical structure of genipin and chlorogenic acid: (a) geniposide; (b) chlorogenic acid.

Geniposide (IUPAC name: methyl (1S,4aS,7aS)-7-hydroxymethyl-1-[(2S,3R,4S,5S,6R)-3,4,5-trihydroxy-6-(hydroxymethyl)oxan-2-yl]oxy-1,4a,5,7a-tetrahydrocyclopenta[c]pyran-4-carboxylate, purity > 98%, catalog number: 171130) and chlorogenic acid (IUPAC name: (1S,3R,4R,5R)-3-[(E)-3-(3,4-dihydroxyphenyl)prop-2-enoyl]oxy-1,4,5-trihydroxycyclohexane-1-carboxylic acid, purity > 98%, catalog number: 171107) are commercially available and were purchased from Shanghai Winherb Medical Technology Co., Ltd. (Shanghai, China). Their respective chemical structures are provided in Figure 1 (Figure 1).

### 2.2. Animals and Treatment

For this study, 24 six-week-old male C57BL/6 mice were purchased from the Shanghai Laboratory Animal Center (Shanghai, China). Animals were maintained at room temperature on a 12 h : 12 h light-dark cycle in the animal center of the Shanghai University of Traditional Chinese Medicine. After acclimation for one week, they were randomly divided into a control group (Con, *n* = 8) and a high-fat diet group (HFHC, *n* = 16). The mice in the HFHC group were fed a high-trans fat, high cholesterol, and high bile salt feed combined with water containing high sugar (45% of the feed energy was derived from fat, Trophic Animal Feed High-Tech Co., Ltd, China, TP23302S, composition of sugar water: 55%fructose + 45%sucrose, concentration 42 g/L), to establish the animal model of NASH mice. Mice in the control group were fed a control diet (10% fat, Trophic Animal Feed high-tech Co., Ltd, China, TP23301S) and fed normal water. After 16 weeks on the respective diets, mice in the HFHC group were randomly but equally divided into the high-trans fat, high-cholesterol, high-bile salt feed combined with high-sugar drinking water group (high-fat and high-sugar feed, HFHC group), and the geniposide and chlorogenic acid combination group (GC group). In the following 5 weeks, the mice were administrated with different drugs by gavage once per day. G (90 mg/kg per day) and C (1.34 mg/kg per day) were dissolved in drinking water, and mice in the HFHC received equal volumes of drinking water. All the animals were sacrificed for tissue collection at the end of the 17th week.

### 2.3. Serum Biochemical Assays

Serum low-density lipoprotein cholesterol (LDL-C) and serum total cholesterol (TC) were measured using a TOSHIBA TBA-40FR Automatic Analyzer (Hitachi, Limited, Tokyo, Japan) with 100 *μ*L of serum liquid. Liver tissue (100 mg) was homogenized in 3 mL of ethanol acetone mixture (1 : 1 in volume). The total hepatic TG was extracted in the medium at 4°C, overnight. After being centrifuged at 1,000 g for 20 min at 4°C, the supernatant was removed for the TG assay according to manufacturer's instructions using Triglycerid Reagent Kit (Dongou Bioengineering, Zhejiang, China). Serum insulin was detected in Ultra Sensitive Mouse Insulin ELISA Kit (18APUMI482A). Activities of serum alanine aminotransferase (ALT), aspartate aminotransferase (AST), and serum glucose (GLU) were quantitated using commercial kits according to the manufacturer's instructions (Nanjing JianCheng Bioengineering Institute, Nanjing, China).

### 2.4. Histological Examination of Hepatic Tissues

For histological examination, the hepatic tissues were fixed in 10% formalin, embedded in paraffin, and cut into 4 *μ*m sections. Tissue sections were then stained with hematoxylin and eosin (H&E) (Nanjing Jiancheng Institute of Bio Engineering, Inc.,). Images were analyzed with a light microscope (Olympus BX40, Tokyo, Japan). Histological liver damage was evaluated via NAFLD activity score (NAS) which includes steatosis, ballooning change, and lobular inflammation. Sirius red (Direct Red 80; Aldrich, WI, US) staining was performed to visualize the pathological changes and collagen deposition. The Sirius red-stained areas were analyzed using the Aperio Image Scope-Pathology Slide Viewing Software (Aperio Image Scope 12.3.3, Leica Biosystems Inc, IL, US). A fibrosis staging system published previously [13] was used to identify the stage of hepatic fibrosis.

### 2.5. Immunocytochemistry

For immunohistochemical staining, formalin-fixed liver sections were deparaffinized and rehydrated by standard protocols and were incubated with specific primary antibodies. Briefly, paraffin-coated liver tissue sections were subjected to conventional dewaxing and rehydration; endogenous peroxidase activity was then blocked using 3% H_2_O_2_ prepared in methanol, and the samples were incubated for 10 min prior to washing three times in PBS. Antigens were then heat repaired in Improved Citrate Antigen Solution (Biotech well, WH1033). Protein blocking was conducted by immersing the samples with 5% bovine serum albumin (BSA; Biotech well, WH1057-1), followed by incubation for 30 minutes at 37°C to block nonspecific background staining. Samples were then washed three times in PBS F4/80 (1 : 100, AbCam, ab111101) and incubated on sections overnight at 4°C. Secondary antibodies used were biotinylated goat anti rabbit IgGs (Biotech Well, WH1057-2), with signal amplification performed using streptavidin-biotin complexes conjugated with peroxidase (SABC, Biotech Well, WH1057-3). Finally, samples were counterstained with hematoxylin.

### 2.6. Detection of Cytokine and Chemokine Protein Levels in Liver Tissue

Cytokine levels in the liver from mice were determined using a multiplexed bead immunoassay using with Luminex Technology (Bio-PlexPro Mouse Cytokine7-plex Panel, Bio-Rad). A bead-based multiplex sandwich immunoassay kit allowed the quantification of the following five cytokines/chemokines in parallel: IL-1*α*, IL-1*β*, IL-6,macrophage chemoattractant protein- (MCP-) 1, and tumor necrosis factor- (TNF-) *α*. For multiplex ELISAs, tissue homogenates were diluted 1 : 2 in Bio-Plex Cell Lysis Buffer (Bio-Rad Laboratories, Hercules, CA) containing complete protease inhibitor cocktail, frozen and thawed, sonicated for 10 s, and centrifuged at 10,000 × g for 10 minutes. The supernatants, containing protein concentrations of 3–4 mg/mL, were diluted 1 : 3 in Bio-Plex sample diluent and used for the multiplex assay according to the manufacturer's recommendations. The beads were analyzed on a Bio-Plex 200 system (Bio-Rad).

### 2.7. Cell Culture and Treatment

Mouse mononuclear macrophages (RAW264.7) were purchased from the Shanghai Institutes for Biological Sciences (China). A combination of geniposide and chlorogenic acid was prepared; 45.4 mg chlorogenic acid (molecular weight: 354.30) and 305.7 mg gardenoside (molecular weight: 388.36) were weighed and resuspended in 1 mL and 900 mL of DMSO, respectively, using aseptic technique. Cells were cultured in Dulbecco's Modified Eagle's Medium supplemented with 10% fetal bovine serum at 37°C in a humidified atmosphere containing 5% CO_2_. Cellular inflammation was induced with 100 ng/mL LPS (076M4013V, Sigma) for 24 h, and equal amount of BSA was added to the control cells. After successfully producing the inflammation model, the cells were incubated in a series of GC concentrations (12.5, 25, and 50 *μ*M) to explore the optimal drug concentration.

### 2.8. Cell Viability Assay

RAW 264.7 cell growth was determined using the CCK 8 assay. Cells (2 × 10^4^) were seeded on 96-well microtiter plates containing 100 *μ*L complete culture medium per well and incubated for 24 h at 37°C. Cultures were transferred into serum-free medium 12 h prior to performing the assay. A range of concentrations of GC reagent (0, 50, 100, 200, 400, and 800 *μ*M) was evaluated. Cells were incubated for further 24 h in an incubator at 37°C with 5% CO_2_. The media was then aspirated, CCK8 added for 2 h, and plates shaken for 10 min. Absorbance was then measured at 490 nm using a microplate reader (Bio-Rad). Cell viability in each group was presented as a percentage of the control.

### 2.9. NO Quantification

The culture media of RAW264.7 was combined with 0.1% sulfanilamide and 0.1% N-(1-naphthyl) ethylenediamine in 5% H3PO4, and absorbance was measured at 540 nm with NaNO2 as a standard to detect NO.

### 2.10. Western Blot Analysis

Cells were first homogenized into cell lysates (RIPA : Phosphatase inhibitors : protease inhibitors = 5 : 1 : 1), which were then run on SDS-acrylamide gels by electrophoresis and transferred to a polyvinylidene difluoride membrane. TBS containing Tween 20 was used as the blocking buffer. The membranes were blocked with 5% BSA for 1 h and then incubated with a primary antibody for TNF-*α* (BS1857, Bioword) at 4°C. The membrane was further reacted with a mixture of 2 mL each of developer A and developer B (P10018M-2, Beyotime Biotechnology) and finally exposed to EZL reagent for chemiluminescence (ChemiScope series, Clinx Science Instruments Co., Ltd.). Glyceraldehyde-3-phosphate dehydrogenase (GAPDH) served as the internal control for total protein loading.

### 2.11. RT-PCR Analysis

Total RNA isolated was subjected to RT-PCR with an RNA PCR kit (RNA extraction: Sangon Biotech (Shanghai) Co., Ltd. B511321; RNA reverse transcription: BioRad, 1708890) for determining mRNA levels of TNF-a, IL-1*β*, IL-6, and iNOS. Nucleotide sequences of the PCR primers are shown in the figure below. In brief, the PCR conditions for reverse transcription are 25°C for 5 min, 46°C for 20 min, and 95°C for 1 min, for 50 cycles. The RT-PCR products were resolved via agarose gel electrophoresis and stained with 1% ethidium bromide.

### 2.12. Statistical Analysis

Data are shown as the mean ± standard deviation (SD). The calculations were performed using GraphPad Prism version 6.0 for Windows (GraphPad Software Inc., San Diego, CA). Two-tailed unpaired Student's *t*-tests were used for comparisons of two groups. The ANOVA multiple comparison test was used for comparing more than two groups. Differences were considered to be statistically significant at *P* < 0.05.

## 3. Results

### 3.1. GC Significantly Decreased Wet Liver Weight and HFHC-Induced High Liver-to-Body Ratio

**Figure 2:**
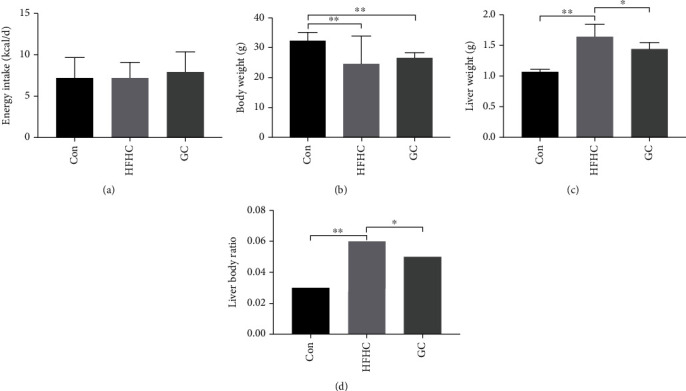
GC significantly decreased wet liver weight and HFHC-induced high liver-to-body ratio. The level of energy intake (a), body weight (b), liver weight (c), and liver body ratio (d) in the mice from each group (*n* = 10). Con: control diet; HFHC: high-fat/carbohydrate/cholesterol/choline diet; GC: geniposide and chlorogenic acid combination. ^∗^*P* < 0.05, ^∗∗^*P* < 0.01.

The energy intake of each group of mice was determined. There was no statistically significant difference between the energy intake of each group (Figure 2(a)). After treatment, the body weight of the mice in the HFHC group was significantly lower than that of the mice in the control group (*P* < 0.05), and there was no statistically significant difference in body weight between the mice of the GC group mice and the HFHC group mice (Figure 2(b)). The wet liver weight and liver-to-body ratio were significantly higher in the HFHC group (*P* < 0.01) and significantly lower in the GC group than in the control group (*P* < 0.05) (Figures 2(c) and 2(d)).

### 3.2. GC Improved Serum Lipids and Glucose Metabolism in HFHC-Induced Mice

**Figure 3:**
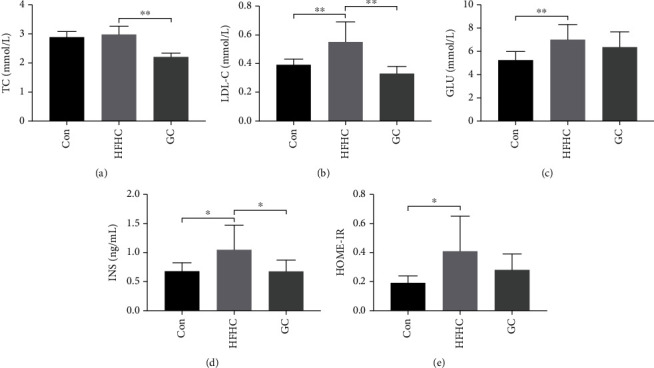
GC improved serum lipids and glucose metabolism in HFHC-induced mice. The content of TC (a), LDL-C (b), GLU (c), INS (d), and HOMA-IR (e) in the mice from each group (*n* = 10). Con: control diet; HFHC: high-fat/carbohydrate/cholesterol/choline diet; GC: geniposide and chlorogenic acid combination. ^∗^*P* < 0.05, ^∗∗^*P* < 0.01.

Compared with the control group, the LDL-C level in the HFHC group was significantly higher (*P* < 0.01). Compared with the HFHC group, Tch and LDL-C levels in the GC group were significantly lower (*P* < 0.01) (Figures 3(a) and 3(b)). Compared with the control group, fasting glucose (GLU), insulin (INS), and HOMA-IR levels in the HFHC group increased significantly (*P* < 0.01 or *P* < 0.05). After GC treatment, INS levels significantly decreased (*P* < 0.05), and GLU and HOMA-IR levels showed a downward trend (Figures 3(c)–3(e)).

### 3.3. GC Reversed Liver Steatosis, Inflammation, and Fibrosis in HFHC-Induced Mice

**Figure 4:**
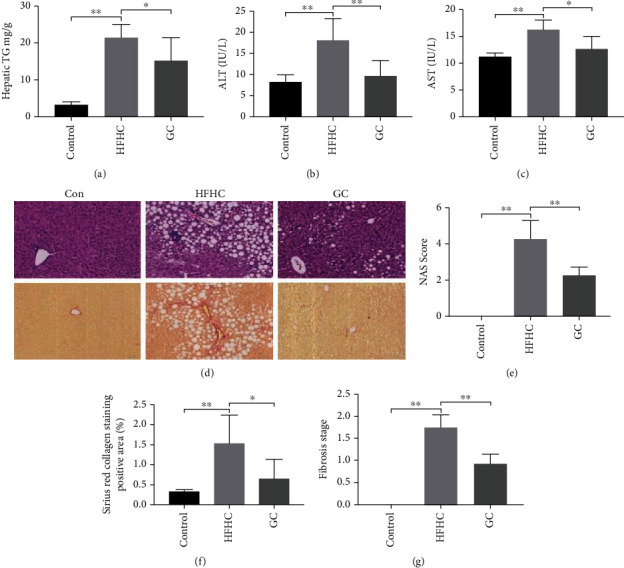
GC reversed liver steatosis, inflammation, and fibrosis in HFHC-induced mice. Hepatic TG content (a), serum ALT and AST activities (b, c), H&E and SR staining on liver sections (200x magnification) (d), NAS score (e), SR collagen-positive staining area (f), and liver fibrosis stage (g). Con: control diet; HFHC: high-fat/carbohydrate/cholesterol/choline diet; GC: geniposide and chlorogenic acid combination. ^∗^*P* < 0.05, ^∗∗^*P* < 0.01.

TG content in the liver tissue was significantly higher in the HFHC group than in the control group (*P* < 0.01). After GC treatment, TG content in the liver tissue significantly decreased (*P* < 0.05) (Figure 4(a)). Serum alanine aminotransferase (ALT) and aspartate aminotransferase (AST) activities were significantly higher in the HFHC group than in the control group (*P* < 0.01). GC significantly downregulated the activities of ALT and AST (*P* < 0.01 or *P* < 0.05) (Figures 4(b) and 4(c)).

H&E staining of liver tissues in the HFHC group showed extensive steatosis (steatosis range > 50%), inflammation, and different degrees of balloon-like degeneration of liver cells. Fatty liver cells were enlarged and appeared round-shaped or oval; the cytoplasm was filled with a large number of fatty vacuoles, and a section of the cytoplasm contained lipid droplets. Macrophages were observed in the liver sinusoids, and a mixture of neutrophils and monocytes was observed in the lobule infiltration of cells. After GC treatment, the fatty degeneration of the liver tissue significantly improved. Balloon-like degeneration and inflammation of liver cells were rare or absent (Figure 4(e)).

The mice in the HFHC group had higher steatosis, inflammation, ballooning degeneration, and NAFLD activity scores (NAS score) than those of mice in the control group (*P* < 0.01). After GC treatment, the steatosis, inflammation, ballooning degeneration, and total NAS score significantly decreased (*P* < 0.01 or *P* < 0.05) (Table 1) (Figure 4(d)).

Sirius scarlet staining results in the HFHC group indicated collagen deposition around the central vein and hepatocytes (Figure 4(e)). Semiquantitative analysis of the stained collagen area was performed, and the stage of liver fibrosis was concurrently evaluated. Compared with the control group, the collagen-positive staining in the HFHC group was significantly increased (*P* < 0.05), and the liver fibrosis stage was severe (*P* < 0.01). After GC treatment, Sirius scarlet staining of the liver tissue was significantly improved, and the collagen staining (*P* < 0.05) and the stage of liver fibrosis were significantly reduced (*P* < 0.01) (Figures 4(f) and 4(g)).

### 3.4. GC Inhibited the Activation of KCs and Decreased Proinflammatory Factor and Chemokine Levels in the Mice Liver Tissue

**Figure 5:**
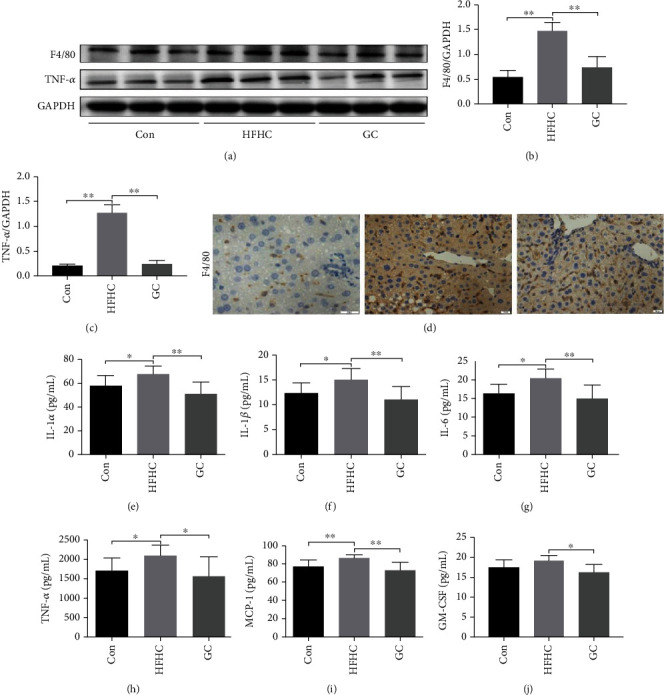
GC inhibited the activation of KCs and decreased proinflammatory factor and chemokine levels in the mice liver tissue. F4/80 and TNF-*α* protein levels in mice were detected by immunoblotting, and the relative expression levels of proteins were corrected by GAPDH (a–c); F4/80 protein expression in mice was detected by immunohistochemical (d). Protein expression of proinflammatory interleukin IL-1*α*, IL-1*β*, IL-6, MCP-1, GM-CSF, and TNF-*α* (e–j). Con: control diet; HFHC: high-fat/carbohydrate/cholesterol/choline diet; GC: geniposide and chlorogenic acid combination. ^∗^*P* < 0.05, ^∗∗^*P* < 0.01.

The expression of F4/80 and tumor necrosis factor-*α* (Tnf-*α*) was significantly higher in the HFHC group than in the control group (*P* < 0.01). In contrast, GC significantly reversed this upregulation (*P* < 0.01) (Figures 5(a)–5(c)).

F4/80 (M1 macrophage and activated Kupffer cell marker) antibody and macrophage IHC staining were used to evaluate macrophage activation and infiltration. F4/80 immunohistochemical results of the HFHC group showed a large amount of F4/80 positive staining in the liver sinusoids around the vein and a small amount of F4/80 positive staining in the liver sinusoids of the control group. The expression of F4/80 was significantly decreased after GC treatment. Immunohistochemistry results of myeloperoxidase (MPO) in the HFHC group showed a large number of neutrophils scattered around the inflammatory foci, and the MPO expression in the liver tissue decreased significantly after GC administration (Figure 5(d)).

GC inhibited the activation of liver macrophages in NASH mice. To further evaluate the effect of GC on the expression of liver macrophage-related inflammatory factors and chemokines, we detected the expression of these cytokine proteins using Luminex. When compared with the control group, expression of proinflammatory interleukin-1*α* (IL-1*α*), IL-1*β*, IL-6, monocyte chemotactic protein 1 (MCP-1), and TNF-*α* was significantly upregulated in HFHC mice. Meanwhile, expression of these inflammatory cytokines was significantly reduced upon treatment with GC (*P* < 0.05 or *P* < 0.01) (Figures 5(e)–5(j)).

### 3.5. GC Inhibited the Activation of RAW264.7 Cells and Decreased the Expression of Proinflammatory Factors and Chemokines

**Figure 6:**
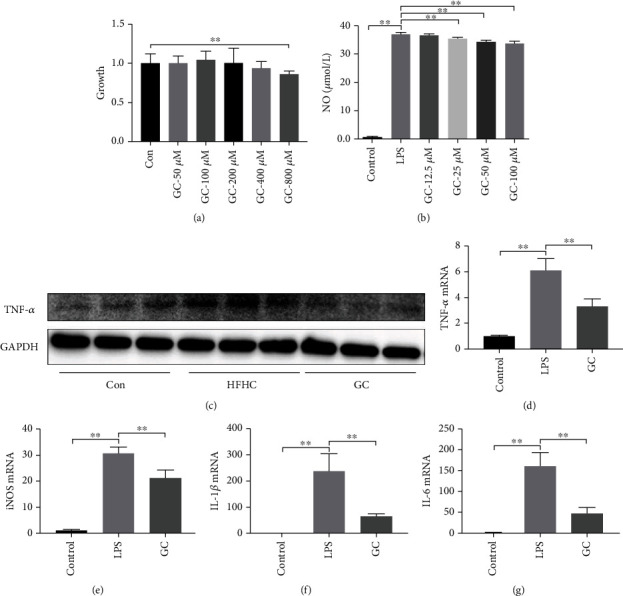
GC inhibited the activation of RAW264.7 cell lines and decreased the expression of proinflammatory factors and chemokines. Toxicity test of GC RAW264.7 cell lines was performed by CCK8 measurement after cells were incubated with different treatment for 24 hours (a). NO content induced by different dosages of GC was test (b). TNF-*α* protein levels in cell were detected by immunoblotting (c). TNF-*α*, iNOS, IL-1*β*, and IL-6 mRNA expression in cells. Con: control diet; HFHC: high-fat/carbohydrate/cholesterol/choline diet; GC: geniposide and chlorogenic acid combination. ^∗^*P* < 0.05, ^∗∗^*P* < 0.01.

After 24 h of adherent growth of RAW264.7 cells, GC was added to the cells at varying concentrations (50, 100, 200, 400, and 800 *μ*M) and incubated for 24 h. Compared with the control group, the proliferation at GC concentrations of 50–400 *μ*M was more than 90%. At a GC concentration of 800 *μ*M, the proliferation was less than 80% (*P* < 0.01), and some cell death was observed (Figure 6(a)).

Following macrophage activation, extracellular l-arginine actively moved into the cell, becoming the main source of NO. NO is the main inflammatory factor in cells. Therefore, we first used NO to detect the inflammatory state of macrophages. The results showed that compared with the control group, the culture contained a large amount of NO after stimulating macrophages with LPS (100 ng/mL), which was statistically significant (*P* < 0.01). After coincubation with GC for 24 h, GC at 12.5, 25, and 50 *μ*M significantly reduced NO levels (*P* < 0.01), showing the best effect at 50 *μ*M. Therefore, a concentration of 50 *μ*M was adopted in our subsequent experiments (Figure 6(b)).

Protein and mRNA levels of TNF-*α* were detected. Compared with the control group, protein and mRNA levels of TNF-*α* in the LPS group were significantly increased (*P* < 0.01). Compared with the LPS group, protein and mRNA levels of TNF-*α* in the GC group decreased significantly (*P* < 0.01) (Figures 6(c) and 6(d)).

mRNA levels of inducible nitric oxide synthase (INOS), IL-1*β*, and IL-6 were also detected. The results showed that the mRNA levels of INOS (*P* < 0.01), IL-1*β* (*P* < 0.01), and IL-6 (*P* < 0.01) were significantly increased in the LPS group compared with the control group. After GC treatment, the mRNA levels of Inos, IL-1*β*, and IL-6 decreased significantly (*P* < 0.01) (Figures 6(e)–6(g)).

## 4. Discussion

In the present study, we found that GC treatment in mice exposed to HFHC diet containing cholesterol, choline, and high-sugar drinking water resulted in the dramatic attenuation of steatohepatitis, including repressed liver steatosis, decreased plasma ALT and AST, and suppression of hepatic inflammation, collagen deposition, and nutritional fibrosis, compared with mice exposed to a control diet. NASH pathohistological features were blocked upon GC treatment in HFHC mice. It is worth noting that GC was effective in improving liver inflammation. Therefore, we speculated that the efficacy in inflammation might be due to the regulation of liver immunity. We aimed to explore whether the suppression of the Kupffer cell activity was one of the underlying mechanisms.

The role of macrophages in NAFLD has been investigated in humans and animal models. In NASH, macrophages can be activated by various stimuli, such as endotoxins, fatty acids, cholesterol, and their metabolites, as well as molecules associated with hepatocyte damage [14]. Human and animal studies provide convincing evidence for the important role for macrophages in NAFLD development and progression. A study on Korean young adults showed increased numbers of KCs in biopsy samples of patients with severe NASH [15]. In disease models, hepatic macrophage infiltration has been demonstrated in mouse dietary models, such as HFD and methionine–choline-deficient (MCD) diet [16, 17], and is also associated with increased age in mice [18]. Liver-resident KCs initiate the inflammatory response and are instrumental in recruiting monocytes to the liver, where they rapidly differentiate into proinflammatory macrophages contributing to NAFLD progression and fibrogenesis [19]. There are several pathways for Kupffer cell activation in NAFLD: via gut-derived endotoxins, such as LPS entering the circulation owing to enhanced intestinal permeability and acting through Toll-like receptors (TLRs); via molecules associated with hepatocyte damage, such as histidine-rich glycoprotein and danger-associated molecular patterns; via free fatty acids through TLRs and adipokines (leptin via the leptin receptor (LEPR) in the adipose tissue); and via cholesterol and its metabolites acting through CD36 and scavenger receptor A (SRA) [20]. KCs then secrete proinflammatory cytokines, such as TNF-*α*, IL-1*β*, and IL-6, to recruit neutrophils and circulating monocytes via the CC-chemokine ligand 2- (CCL2-) CC-chemokine receptor 2 (CCR2) interaction [17]. Monocytes differentiate into proinflammatory macrophages to further amplify hepatic inflammation and contribute to fibrogenesis via stimulating hepatic stellate cells by transforming growth factor-*β* (TGF-*β*), facilitating their transformation into activated myofibroblasts upon extracellular matrix deposition [21]. However, conventional high-fat diets are difficult to successfully induce the above significant NASH characteristics [22]. Addition of high cholesterol and high bile salts is added to the diet leads to the induction of more obvious hepatocyte steatosis, hepatocyte ballooning, inflammation, and liver fibrosis. Another advantage of the model is that it is similar to the pathological characteristics of human NASH [23]. Studies have shown that, compared with the control group, mice fed HFD with high cholesterol and high bile salts lose 9% of their body weight and have reduced epididymal fat [24]. The diet in our study contained 2% cholesterol (conventional HFD contains 0.5–1% cholesterol) and 0.5% bile salt (HFD does not generally contain bile salt). In addition, fructose and sucrose were added to drinking water to promote disease progression. All of these successfully induced NASH accompanied by a certain degree of liver fibrosis. Basing on our data, mice continuously fed with HFHC for 16 weeks developed NASH, which replicates many pathophysiological features of human NASH; infiltration of F4/80+ macrophages is observed, as well as the activation of KCs with increased expression of proinflammatory cytokines including IL-6, TNF-*α*, IL-1*β*, IL-1*α*, MCP-1, and GM-CSF to further enhance the inflammatory reaction. These similar results indicate that macrophages have been activated in this model. Furthermore, the activation of KCs has many effects on the further development of NASH.

With progression of steatosis and balloon-like degeneration of liver cells, KCs secrete chemokines, including CCL2 and MCP-1, through the infiltration of monocytes, thereby increasing the production of macrophages. Next, through the feedback mechanism, the secretion of a large number of proinflammatory cytokines, such as IL-1*β* and TNF-*α*, is increased, which further promotes liver steatosis, inflammation, and liver fibrosis. The infiltration of KCs is also observed in NAFLD or NASH models induced by an HFD or MCD diet, and it tends to increase with age. Further studies have shown that if the infiltration of macrophages is inhibited in the progression of NAFLD, the occurrence of hepatocyte steatosis can be inhibited. A study on HFD-fed mice with chlorophosphate (inhibiting macrophage infiltration) revealed that after liver macrophages are depleted, liver fat is reduced [25]. After IL-1*β*-dependent PPAR*α* is inhibited, the protective effect caused by the decrease in KC levels is reduced. Reduced utilization of KCs can reduce the secretion of inflammatory factors, inflammation, oxidative damage of liver cells, and the expression of liver fibrosis genes, such as TGF-*β* and COL-I [26]. Considering the essential role of macrophages in fatty liver disease, different mechanisms and events in their recruitment, activation, and action might be suitable targets for therapeutic intervention. Meanwhile, natural active ingredients extracted from herbs or foods have a good therapeutic effect on NASH. For example, EGCG extracted from green tea [27] and caffeine [28] have a variety of pharmacological effects on NASH. In addition to the effects on KCs, geniposide and chlorogenic acid have also been reported to improve NAFLD by upregulating the expression of PPAR-*α* [29] and GLP-1R [30] in hepatocytes. At present, the understanding of the pathophysiology of NAFLD provides a practical theoretical basis for the combined treatment of the disease [31]. GC combination therapy shows the preliminary idea of combination therapy to a certain extent and also provides a promising option for natural active ingredients to treat NAFLD.

There are some limitations and shortcomings to our study. This animal model causes weight loss, mainly because bile salts promote the absorption of cholesterol, induce cholesterol metabolism, and participate in bile acid metabolism. This has brought some negative effects on the pharmacodynamic evaluation, but the immune disorder of this model is relatively typical. Monocytes can be polarized toward M1-type or M2-type (classically and alternatively activated, respectively) macrophages in vitro. Due to the limitations of flow cytometry, we only used F4/80 to mark the macrophages and did not carry out further studies on the M-type polarization of liver macrophages. We will aim to investigate this further in future studies.

## 5. Conclusion

In summary, GC has a good curative effect on steatosis, inflammation, fibrosis, and insulin resistance in NASH. In terms of innate immunity, GC may use macrophages to improve inflammation. GC could be a therapeutic agent for NASH via inhibition of KC activation, and the underlying mechanism of these effects warrants further research.

## Figures and Tables

**Table 1:** Comparison of NAS scores in liver tissues of mice in each group ($\bar{X}\pm S$).

Group	*n*	Steatosis	Inflammation	Ballooning change	NAS store
Con	8	0.00 ± 0.00	0.00 ± 0.00	0.00 ± 0.00	0.00 ± 0.00
HFHC	8	1.63 ± 0.52^∗∗^	1.75 ± 0.46^∗∗^	0.88 ± 0.64^∗∗^	4.25 ± 1.04^∗∗^
GC	8	1.13 ± 0.35^#^	0.75 ± 0.46^#^	0.25 ± 0.46^#^	2.25 ± 0.46^##^

Compared with the Con group, ^∗∗^*P* < 0.01; compared with the HFHC group, ^#^*P* < 0.05, ^##^*P* < 0.01.

## Data Availability

These are commercial confidentiality.

## Abbreviations

NAFLD: Nonalcoholic fatty liver disease
NASH: Nonalcoholic steatohepatitis
GC: Geniposide and chlorogenic acid
QHD: Qushi Huayu Decoction
MCP-1: Monocyte chemotactic protein 1
TNF-*α*: Tumor necrosis factor-*α*
IL: Interleukin
GM-CSF: Granulocyte-macrophage colony stimulating factor
TG: Triglyceride
TC: Total cholesterol
FFA: Free fatty acid
FBG: Fasting blood glucose
IR: Insulin resistance
ALT: Alanine aminotransferase
AST: Aspartate transaminase
HFD: High-fat diet
T2DM: Type 2 diabetes mellitus.
